# Embodied Sensory Deprivation Hypothesis (ESDH): An Evolutionary Model of Sensory Malnutrition Linking Interoceptive Dysregulation, Anxiety, and Disorders of Gut-Brain Interaction

**DOI:** 10.7759/cureus.113925

**Published:** 2026-08-04

**Authors:** Alexander Bertuccioli, Chiara Maria Palazzi, Giordano Bruno Zonzini, Annalisa Belli, Francesco Di Pierro, Marco Cardinali

**Affiliations:** 1 Biomolecular Sciences Department, University of Urbino Carlo Bo, Urbino, ITA; 2 Coordination Department, Microbiota International Clinical Society, Torino, ITA; 3 Administration Department, Microbiota International Clinical Society, Torino, ITA; 4 Management Department, Microbiota International Clinical Society, Torino, ITA; 5 Scientific and Research Department, Velleja Research, Milan, ITA; 6 Medicine and Technological Innovation Department, University of Insubria, Varese, ITA; 7 Internal Medicine Department, Azienda Sanitaria Territoriale 1 (AST1), Pesaro, ITA

**Keywords:** disorders of gut-brain interaction (dgbi), embodied sensory deprivation, interoception, predictive coding, sensory malnutrition

## Abstract

The human nervous system evolved in environments characterized by continuous exposure to rich, multimodal bodily inputs, including locomotion, manual activity, physical effort, social touch, sexual contact, environmental variability, thermal fluctuations, and complex proprioceptive challenges. In contrast, modern lifestyles have progressively reduced many of these embodied sensory exposures through sedentarism, automation, climate-controlled environments, cushioned footwear, reduced manual labor, digital socialization, and diminished physical contact. Here, we propose the Embodied Sensory Deprivation Hypothesis (ESDH), an evolutionary and neurophysiological framework suggesting that a chronic reduction in the quantity, diversity, and variability of biologically meaningful sensory inputs may impair the calibration of interoceptive, proprioceptive, tactile, thermosensory, and autonomic regulatory systems. Contemporary models of predictive coding, active inference, and allostasis describe the brain as a prediction-generating organ that continuously regulates the body by integrating incoming sensory signals with prior expectations. Within this framework, chronic sensory impoverishment may increase physiological uncertainty, amplify prediction errors, and contribute to maladaptive autonomic, emotional, and somatic responses.

We further suggest that the cumulative loss of embodied sensory experiences may be conceptualized as a form of sensory malnutrition, reflecting a mismatch between the sensory ecology in which the human nervous system evolved and the sensory conditions typical of modern industrialized societies. We hypothesize that this mismatch may contribute to anxiety-related conditions, somatic hypervigilance, psychosomatic symptom expression, and Disorders of Gut-Brain Interaction (DGBI), all of which involve altered interoceptive processing, autonomic dysregulation, and disrupted body-brain communication. At present, ESDH should be considered a hypothesis-generating model rather than a defined clinical entity. However, if future observational and interventional studies identify a reproducible phenotype characterized by chronic embodied sensory impoverishment, interoceptive dysregulation, autonomic instability, anxiety, somatic amplification, and responsiveness to sensory enrichment interventions, this construct may evolve into a formal clinical syndrome, provisionally termed Embodied Input Deficiency Syndrome (EIDS). The ESDH framework offers a novel integrative perspective linking interoception, predictive neuroscience, evolutionary medicine, psychosomatic medicine, and gut-brain interaction research. By reframing modern sensory impoverishment as a potential contributor to body-brain dysregulation, it generates testable predictions for future clinical, translational, and interventional studies.

## Introduction and background

Introduction

The human nervous system evolved within environments characterized by continuous exposure to diverse and biologically meaningful sensory experiences. Throughout most of human evolutionary history, daily survival depended upon locomotion, manual activity, environmental exploration, physical effort, social interaction, tactile contact, sexual behavior, thermal variability, and constant adaptation to changing physical surroundings. Consequently, the brain developed not as an isolated computational organ but as a predictive system continuously shaped by dynamic interactions between the body and the environment.

Contemporary neuroscience increasingly recognizes that cognition, emotion, and self-awareness emerge from ongoing bidirectional communication between the brain and the body. Within this framework, interoception, the sensing, integration, and interpretation of internal bodily signals, plays a central role in emotional regulation, autonomic control, adaptive behavior, and subjective experience [1-5]. In parallel, predictive coding, active inference, and free energy models propose that the brain continuously generates predictions about bodily states and updates these predictions through incoming sensory information [1-5]. Rather than passively receiving sensory inputs, the nervous system actively constructs models of physiological reality and continuously refines them through interaction with the body and the external environment.

The quality of this predictive process depends upon the availability of reliable sensory information. Under physiological conditions, diverse streams of sensory input, including proprioceptive, tactile, vestibular, thermosensory, cardiorespiratory, and visceral signals, provide continuous feedback that allows the nervous system to calibrate its predictions and maintain adaptive regulation. In this perspective, bodily sensations are not merely consequences of physiological processes; they are fundamental components of the regulatory architecture supporting emotional stability, autonomic flexibility, and behavioral adaptation.

Modern industrialized societies have profoundly altered the sensory environments in which humans live. Mechanized transportation, prolonged sitting, climate-controlled environments, digital communication, cushioned footwear, reduced manual labor, and diminished physical contact have collectively reduced exposure to many forms of embodied sensory stimulation that were historically ubiquitous. While these environmental changes have undoubtedly produced substantial benefits, they may also have created conditions that differ significantly from those under which human neurophysiology evolved.

Evolutionary medicine has long proposed that many contemporary chronic disorders emerge from a mismatch between ancestral biological adaptations and modern environmental conditions [6,7]. Examples include obesity, metabolic syndrome, circadian disruption, physical inactivity-related diseases, and other chronic noncommunicable disorders. However, relatively little attention has been directed toward the possibility that a similar mismatch may exist at the level of sensory physiology and body-brain regulation.

We propose that the modern environment may expose individuals to a form of chronic embodied sensory impoverishment, characterized not by the complete deprivation of any single sensory modality but rather by a progressive reduction in the quantity, diversity, and variability of biologically meaningful bodily inputs. This reduction may affect multiple sensory domains simultaneously, including proprioceptive, mechanosensory, tactile, thermosensory, vestibular, and socially mediated bodily experiences.

**Figure 1:**
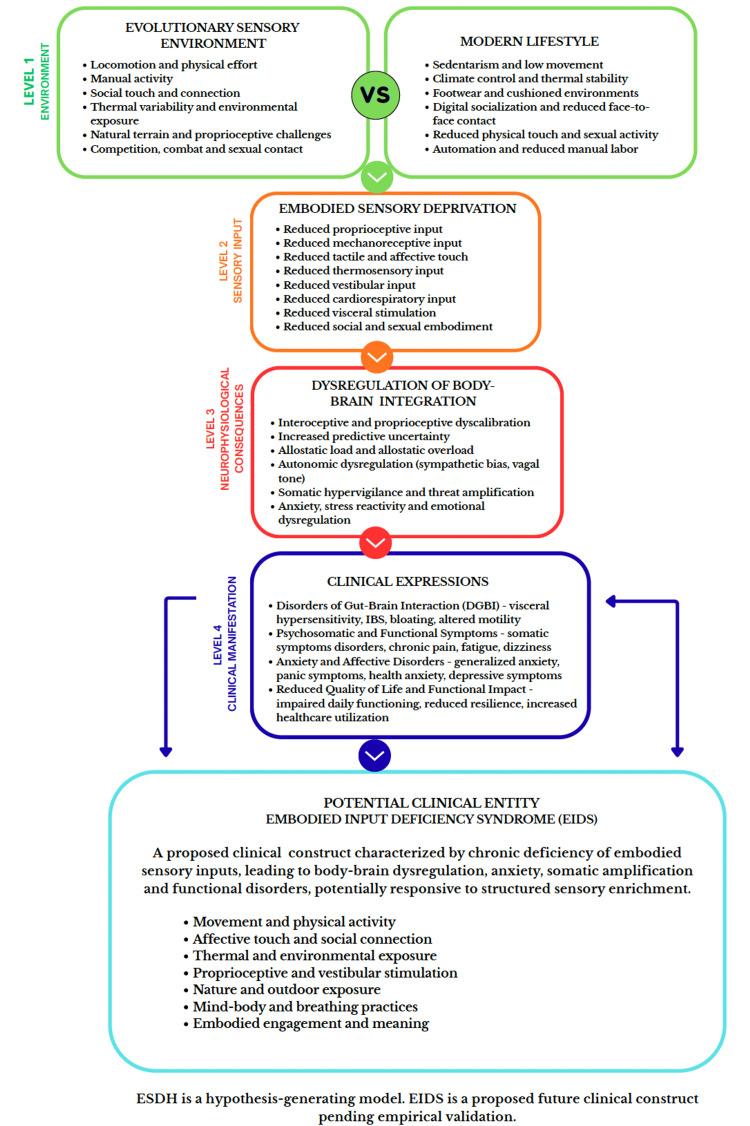
Conceptual framework of the Embodied Sensory Deprivation Hypothesis (ESDH). Modern industrialized environments may reduce the quantity, diversity, and variability of biologically meaningful embodied sensory inputs. This sensory impoverishment is proposed to contribute to sensory malnutrition, impaired interoceptive calibration, increased physiological uncertainty, elevated allostatic burden, and autonomic dysregulation, thereby increasing vulnerability to anxiety-related symptoms, somatic hypervigilance, psychosomatic symptom expression, and Disorders of Gut-Brain Interaction. The proposed clinical construct of the Embodied Input Deficiency Syndrome remains hypothetical and requires empirical validation. This image was manually created by the authors using Canva. No generative artificial intelligence tools, AI-generated elements, or AI-based image modification tools were used. IBS: irritable bowel syndrome

This article introduces the Embodied Sensory Deprivation Hypothesis (ESDH) as a conceptual and hypothesis-generating framework examining whether chronic sensory impoverishment may be associated with interoceptive dysregulation, anxiety-related symptoms, psychosomatic symptom expression, and Disorders of Gut-Brain Interaction (DGBI). It also outlines a research pathway for investigating whether a reproducible multidimensional phenotype associated with chronic embodied sensory impoverishment can be identified. For heuristic purposes, such a provisional research construct is referred to as Embodied Input Deficiency Syndrome (EIDS). At present, EIDS should not be interpreted as an established clinical syndrome or diagnostic entity, and its validity, boundaries, specificity, and clinical utility remain to be determined through observational, mechanistic, and interventional studies. The proposed relationships are potentially reciprocal and multidirectional and may be influenced by biological, developmental, psychological, social, and lifestyle factors. The overall conceptual framework is summarized in Figure 1.

Approach to literature identification and conceptual synthesis

This manuscript is a conceptual hypothesis paper informed by a structured narrative review of the interdisciplinary literature. Relevant publications were identified through searches of PubMed, Scopus, and Web of Science using combinations of terms related to interoception, predictive processing, active inference, allostasis, sensory deprivation and enrichment, affective touch, physical activity, anxiety, psychosomatic symptoms, and Disorders of Gut-Brain Interaction. Publications were considered for inclusion when they addressed at least one of these predefined conceptual domains. Both empirical studies and theoretical or review articles were considered, including findings that supported, qualified, or challenged the proposed framework. Reference lists of key reviews and articles were also examined. Because the objective was conceptual integration rather than exhaustive evidence synthesis, no formal risk-of-bias assessment, meta-analysis, or statistical pooling was conducted.

Theoretical background: Interoception, prediction, active inference, and allostasis

Interoception refers to the sensing, integration, and interpretation of signals arising from within the body, including cardiovascular, respiratory, gastrointestinal, metabolic, immune, thermal, and nociceptive information [5]. Although traditionally considered a sensory process, interoception is increasingly recognized as a fundamental component of emotional regulation, self-awareness, autonomic control, and adaptive behavior [1-5]. Rather than simply informing the brain about the current state of the organism, interoceptive signals actively participate in the construction of subjective experience and physiological regulation.

Contemporary theories of brain function have progressively shifted away from purely reactive models of perception. According to predictive coding frameworks, the brain does not passively receive sensory information but continuously generates predictions regarding both external and internal bodily states [1-4]. Incoming sensory signals are compared to these predictions, and discrepancies between expected and observed states generate prediction errors that drive model updating and adaptive responses.

Within this perspective, interoception can be understood as a predictive process in which the brain continuously estimates the physiological condition of the organism and adjusts regulatory responses accordingly [1]. The subjective experience of bodily sensations therefore emerges from the dynamic interaction between top-down predictions and bottom-up sensory signals rather than from sensory input alone.

These concepts have been further developed through active inference theory and the free energy principle proposed by Friston [3]. According to this framework, biological systems maintain their integrity by minimizing uncertainty and reducing discrepancies between predicted and actual states. Sensory information plays a critical role in constraining predictions and enabling adaptive engagement with both the internal and external environment. In this context, uncertainty is not merely a cognitive phenomenon but a biological challenge that must be continuously regulated to maintain physiological stability.

Owens et al. extended these concepts to clinical neuroscience through the notion of interoceptive inference, proposing that disturbances in the processing of bodily signals may contribute to a wide range of psychiatric and psychosomatic disorders [4]. Alterations in the generation, updating, or interpretation of bodily predictions may lead to maladaptive physiological and emotional responses, particularly when sensory information is ambiguous, inconsistent, or insufficient.

These ideas converge with contemporary models of allostasis. Unlike classical homeostasis, which emphasizes the correction of physiological deviations after they occur, allostasis refers to the anticipatory regulation of bodily systems in response to predicted demands [2]. Through allostatic mechanisms, the brain continuously allocates resources, coordinates autonomic responses, and prepares the organism for future challenges before significant physiological disturbances emerge.

Importantly, successful allostatic regulation depends upon accurate and reliable sensory information. The nervous system must continuously receive signals from the body to refine predictions, estimate physiological requirements, and adjust regulatory responses. When bodily signals are sufficiently rich, diverse, and contextually meaningful, predictive models can be calibrated effectively, and physiological uncertainty remains relatively low.

Conversely, when sensory information becomes impoverished, monotonous, or insufficiently variable, the nervous system may encounter increasing difficulty in accurately modeling bodily states. Reduced sensory input may limit the precision of prediction updating, thereby increasing uncertainty and potentially elevating the allostatic burden required to maintain adaptive regulation. Although this possibility has not been directly investigated as a unified phenomenon, it is conceptually consistent with predictive coding, active inference, and allostatic frameworks [1-4].

The Embodied Sensory Deprivation Hypothesis builds upon these theoretical foundations. Specifically, we propose that chronic reduction in the diversity and richness of embodied sensory inputs may impair the calibration of predictive body-brain models. This impairment may contribute to increased physiological uncertainty, altered interoceptive processing, and maladaptive autonomic responses. Importantly, this proposition remains hypothetical and should be viewed as a testable extension of existing theoretical frameworks rather than an established mechanism.

Within this model, anxiety, somatic hypervigilance, and certain forms of psychosomatic symptom expression may emerge not solely from excessive sensory stimulation or cognitive dysfunction but also from inadequate embodied sensory calibration. In other words, the nervous system may become dysregulated not only by receiving too much information but also by receiving too little meaningful information from the body.

## Review

The concept of embodied sensory deprivation

Embodied sensory deprivation is defined here as a chronic reduction in the quantity, diversity, and variability of biologically meaningful sensory inputs required for optimal neurophysiological regulation.

This construct differs substantially from the traditional concept of sensory deprivation, which generally refers to the partial or complete removal of a major sensory modality such as vision, hearing, or touch. In contrast, the Embodied Sensory Deprivation Hypothesis does not describe the absence of a single sensory channel. Rather, it refers to a cumulative and progressive reduction in multiple forms of embodied sensory experience that normally contribute to body-brain communication.

From this perspective, the nervous system may depend not only upon the presence of sensory signals but also upon their diversity, variability, and ecological relevance. Throughout evolutionary history, humans were continuously exposed to changing bodily experiences generated by movement, environmental interaction, social contact, physical effort, and adaptation to fluctuating conditions. Such experiences likely provided a continuous stream of information allowing the nervous system to refine predictive models, regulate autonomic responses, and maintain adaptive behavioral flexibility.

In modern environments, however, many of these sensory experiences may be substantially reduced. Individuals may spend prolonged periods seated, move across highly predictable surfaces, interact primarily through digital media, experience limited thermal variability, and engage in fewer forms of manual, exploratory, or physically demanding activity. Although none of these factors constitutes complete sensory deprivation, their cumulative effect may produce a progressive reduction in embodied sensory richness.

Within the ESDH framework, embodied sensory deprivation may involve multiple sensory domains simultaneously, as summarized in Table 1.

**Table 1:** Sensory domains involved in embodied sensory deprivation and examples of embodied sensory inputs. Authors’ original conceptual synthesis informed by the literature on interoception, predictive processing, affective touch, and physical activity [1-5,8].

Sensory domain	Examples of embodied sensory inputs
Proprioceptive inputs	Locomotion; postural adjustments; muscular contractions; resistance against gravity; balance and coordination challenges
Mechanosensory inputs	Contact with natural surfaces; manual manipulation of objects; weight-bearing activities; environmental interaction; tactile exploration
Affective and social touch	Interpersonal touch; affectionate physical contact; caregiving touch; social bonding behaviors; sexual contact
Thermosensory inputs	Exposure to environmental temperature fluctuations; adaptation to heat and cold; seasonal variability; natural climatic conditions
Vestibular inputs	Changes in body position; acceleration and deceleration; navigation through complex environments; spatial orientation challenges
Cardiorespiratory inputs	Physiological responses to exercise; fluctuations in respiratory rate; cardiovascular adaptation to effort; autonomic adjustments during movement
Visceral inputs	Gastrointestinal sensations; respiratory sensations; cardiovascular sensations; metabolic and energetic fluctuations

Importantly, the hypothesis does not suggest that the reduction of any single sensory domain is sufficient to produce pathology. Rather, we propose that the simultaneous reduction of multiple embodied sensory channels may generate a cumulative state of sensory impoverishment capable of influencing predictive regulation and allostatic functioning.

A useful analogy can be drawn from nutritional physiology: just as cumulative inadequacy across multiple nutrients can impair physiological function, cumulative reduction across multiple sensory domains may alter body-brain regulation.

For this reason, we propose that embodied sensory deprivation may be conceptualized as a form of sensory malnutrition, characterized not by the complete absence of stimulation but by insufficient exposure to the diversity of sensory experiences for which the nervous system evolved.

At present, the concept of sensory malnutrition remains theoretical. However, it provides a potentially useful framework through which seemingly unrelated modern phenomena, including sedentarism, reduced social touch, environmental standardization, digitalization, and physical inactivity, may be interpreted as components of a broader sensory ecosystem influencing human physiology and behavior.

Within the ESDH model, sensory malnutrition represents the putative initiating condition from which subsequent alterations in interoceptive calibration, allostatic regulation, autonomic functioning, and symptom perception may emerge.

Modern environment, evolutionary mismatch, and sensory nutrition

Modern industrialized societies have produced unprecedented improvements in safety, comfort, sanitation, communication, and life expectancy. However, these advances have also transformed many aspects of the sensory environments in which humans live. Compared to the conditions under which human neurophysiology evolved, contemporary lifestyles are characterized by reduced physical effort, increased automation, prolonged sedentary behavior, diminished environmental variability, and progressively lower exposure to diverse embodied sensory experiences.

Evolutionary medicine proposes that many chronic disorders arise from a mismatch between ancestral biological adaptations and modern environmental conditions [6,7]. Human physiology evolved within environments requiring frequent locomotion, manual activity, environmental exploration, social interaction, exposure to climatic fluctuations, and continuous adaptation to changing sensory conditions. In contrast, modern lifestyles often provide highly standardized, predictable, and physically passive environments.

Examples of this mismatch have already been described in relation to obesity, metabolic syndrome, cardiovascular disease, circadian disruption, and physical inactivity-related disorders [6,7]. We propose that a similar mismatch may exist at the level of sensory physiology.

Historically, the human nervous system was continuously exposed to a broad spectrum of sensory information generated through movement, social contact, environmental exploration, and interaction with complex physical surroundings. Uneven terrain, changing temperatures, variable weather conditions, manual labor, interpersonal touch, competition, cooperation, and sexual behavior all contributed to a rich and continuously changing sensory landscape. These experiences were not optional additions to daily life but represented the normal biological context within which neural regulatory systems evolved.

By contrast, modern environments may substantially reduce sensory variability. Individuals may spend large portions of the day seated, travel using mechanized transportation, interact primarily through digital devices, live within climate-controlled buildings, walk on uniform surfaces, and experience reduced physical contact with others. While each of these changes may appear minor when considered individually, their cumulative effect may result in a profound alteration of the sensory ecosystem surrounding the human organism.

Within this framework, we propose the concept of sensory nutrition.

Sensory nutrition refers to the quantity, diversity, and variability of biologically meaningful sensory experiences required to support optimal body-brain regulation. Just as metabolic systems require an adequate intake of nutrients to maintain physiological homeostasis, neural regulatory systems may require adequate exposure to embodied sensory inputs to maintain predictive accuracy, autonomic flexibility, and allostatic stability.

The concept of sensory nutrition does not imply that all sensory stimulation is beneficial. Excessive, chaotic, or threatening sensory environments may themselves be maladaptive. Rather, sensory nutrition refers specifically to sensory experiences that are evolutionarily familiar, biologically relevant, and capable of supporting adaptive interaction between the organism and its environment.

Examples of potentially relevant components of sensory nutrition and sensory malnutrition are summarized in Table 2.

**Table 2:** Components of sensory nutrition and sensory malnutrition. Authors’ original conceptual framework informed by the evolutionary mismatch theory and literature on affective touch and physical activity [6-9].

Dimension	Sensory nutrition	Sensory malnutrition
Movement	Walking, running, climbing, and resistance exercise	Prolonged sitting and low movement variability
Touch	Affectionate touch, social contact, and caregiving touch	Reduced interpersonal contact and digital-only interaction
Environment	Outdoor exposure, natural settings, and variable terrain	Indoor life, climate-controlled environments, and uniform surfaces
Thermal input	Seasonal variability and moderate cold/heat exposure	Constant artificial temperature
Manual activity	Gardening, craftsmanship, and object manipulation	Automation and limited hands-on activity
Interoceptive challenge	Exercise-induced heart rate and breath and effort changes	Reduced physiological variability

Within this model, insufficient exposure to these experiences may result in a condition that can be described as sensory malnutrition.

Sensory malnutrition is defined as a chronic state in which the quantity, diversity, or variability of embodied sensory inputs falls below the level required to support optimal neurophysiological regulation. Importantly, sensory malnutrition does not imply the complete absence of stimulation. Indeed, modern individuals may be exposed to enormous amounts of visual, informational, and digital stimulation while simultaneously experiencing a deficit of embodied sensory experiences.

In this regard, the modern sensory environment may be characterized by a paradoxical combination of sensory excess and sensory deficiency. Information-rich but embodiment-poor environments may expose the nervous system to large quantities of cognitive input while simultaneously depriving it of many of the sensory signals that historically contributed to predictive calibration and physiological regulation. Mechanistically, this paradox may be understood in terms of limited computational and attentional resources. Contemporary predictive-processing models suggest that the brain continuously allocates precision and attentional resources to update generative models of both external and internal states. Modern industrialized environments often expose individuals to high-salience, low-ecological exteroceptive stimulation, including rapid visual streams, continuous digital alerts, and fragmented informational demands. Such stimulation may increase cognitive and exteroceptive load without providing the embodied proprioceptive, interoceptive, tactile, thermosensory, and vestibular inputs required for body-brain calibration.

Therefore, digital and informational excess should not be considered equivalent to sensory enrichment. Rather, external cognitive overload may compete with the processing of subtle bottom-up bodily signals, potentially reducing the precision with which visceral and proprioceptive prediction errors are detected, integrated, and used to update interoceptive models. In this sense, modern sensory excess and embodied sensory deficiency may coexist: the nervous system may be overstimulated at the exteroceptive-cognitive level while remaining undernourished at the embodied physiological level [1-5].

We hypothesize that sensory malnutrition may represent the environmental substrate underlying embodied sensory deprivation. Through progressive reduction of embodied sensory diversity, sensory malnutrition may contribute to alterations in predictive body-brain modeling, increased physiological uncertainty, and elevated allostatic burden. Although this proposition remains speculative, it is consistent with the evolutionary mismatch theory and with contemporary models emphasizing the importance of sensory information for adaptive regulation [1-7].

Within the ESDH framework, sensory malnutrition represents the proposed bridge between modern environmental conditions and subsequent alterations in interoception, autonomic regulation, emotional processing, and symptom perception. It therefore provides a conceptual mechanism through which environmental change may influence vulnerability to anxiety-related conditions, psychosomatic symptoms, and Disorders of Gut-Brain Interaction.

Affective touch, physical activity, and embodied regulation

Among the various forms of embodied sensory experience, affective touch and physical activity represent two of the most biologically relevant sources of sensory information available to the human nervous system. Both provide continuous streams of proprioceptive, mechanosensory, autonomic, and emotional signals that contribute to body-brain communication and physiological regulation.

Touch is not merely a means of detecting physical contact with the environment. Contemporary neuroscience distinguishes between discriminative touch, which supports object recognition and spatial localization, and affective touch, which contributes to emotional processing, social bonding, and autonomic regulation [8]. Specialized C-tactile afferents appear particularly involved in the processing of affective touch and project through neural pathways closely associated with interoceptive and emotional networks [8].

From an evolutionary perspective, affective touch likely contributed to the regulation of social cohesion, caregiving, attachment, and cooperative behavior. Physical contact between conspecifics is observed across numerous mammalian species and appears deeply integrated into neurobiological systems involved in stress regulation, safety perception, and emotional well-being.

Recent evidence supports the importance of touch for both mental and physical health. A large systematic review and multivariate meta-analysis demonstrated that touch interventions are associated with improvements in psychological well-being and reductions in anxiety, depression, and pain-related outcomes [9]. Importantly, these effects were observed across multiple populations and intervention types, suggesting that touch may represent a broadly relevant regulatory mechanism rather than a context-specific phenomenon.

A compelling demonstration of the biological importance of embodied sensory contact comes from early human development. Historical observations by Spitz [10] and Spitz et al. [11] described how infants raised in institutional settings, despite receiving adequate nutrition and medical care, frequently exhibited impaired development, increased susceptibility to illness, and higher mortality when deprived of consistent maternal contact, a phenomenon termed hospitalism and maternal deprivation. More recently, Kangaroo Mother Care (KMC), based on prolonged skin-to-skin contact between caregiver and infant, has been associated with reductions in mortality, severe infections, and physiological instability among preterm and low-birth-weight neonates [12-15]. Although neonatal findings cannot be directly extrapolated to adult physiology, they provide a proof of concept that embodied sensory contact may function as a biologically relevant regulatory input rather than merely a source of comfort. Historical experimental work by Harlow in rhesus monkeys provided influential evidence that attachment-related behavior was strongly directed toward a soft surrogate providing contact comfort, even when nourishment was supplied by a separate wire surrogate [16]. Although these animal findings cannot be directly generalized to human development, they suggested that tactile comfort and attachment-related contact may serve regulatory functions extending beyond nutrition.

Additional evidence emerged during the COVID-19 pandemic, when restrictions on physical interaction created unprecedented opportunities to observe the effects of reduced interpersonal contact. Individuals experiencing social touch deprivation frequently reported increased loneliness, reduced well-being, and greater desire for physical contact, highlighting the potential importance of touch as a component of normal psychophysiological regulation [17].

Within the ESDH framework, affective touch may therefore be conceptualized as a form of sensory nutrition. Reduced exposure to meaningful interpersonal contact may represent one component of a broader state of embodied sensory impoverishment capable of influencing emotional regulation and physiological stability.

Physical activity provides a second major source of embodied sensory information. Exercise and movement continuously generate proprioceptive, vestibular, mechanosensory, cardiorespiratory, and interoceptive signals. Every change in posture, muscle contraction, respiratory adjustment, or cardiovascular response contributes to the stream of information through which the nervous system monitors and regulates the body.

While the psychological and metabolic benefits of physical activity are well-established, growing attention has been directed toward its role in shaping interoceptive processing [18]. Physical activity exposes the nervous system to dynamic fluctuations in heart rate, respiration, muscular tension, energy expenditure, and recovery, potentially enhancing the accuracy and adaptability of body-brain communication.

Wallman-Jones et al. proposed that physical activity may influence interoceptive processing by increasing exposure to bodily signals and promoting more effective integration of physiological information [18]. Although the precise mechanisms remain incompletely understood, this perspective aligns closely with the theoretical foundations of ESDH.

From this viewpoint, physical inactivity may represent more than the absence of exercise. It may also constitute a reduction in sensory diversity. Sedentary behavior limits exposure to movement-related signals, reduces physiological variability, and may diminish opportunities for the nervous system to update predictive models of bodily states.

The combination of reduced physical activity and reduced interpersonal touch is particularly relevant. Both phenomena have become increasingly common in modern societies and may act synergistically to decrease embodied sensory richness. One reduces movement-derived sensory information, while the other reduces socially mediated tactile input. Together, they may contribute to a progressive reduction in the diversity of sensory experiences available for body-brain calibration.

Importantly, the present hypothesis does not claim that reduced touch or reduced movement independently causes anxiety, psychosomatic symptoms, or DGBI. Rather, these factors are proposed as components of a broader sensory ecosystem. Within this ecosystem, multiple forms of embodied sensory reduction may accumulate over time, potentially contributing to increased physiological uncertainty and altered regulatory processes.

We therefore hypothesize that affective touch and physical activity may represent two central pillars of sensory nutrition. Their reduction may constitute important contributors to embodied sensory deprivation, whereas their restoration may represent key components of future sensory enrichment strategies aimed at improving body-brain regulation.

From embodied sensory deprivation to anxiety: The role of interoceptive dysregulation

Anxiety disorders are traditionally conceptualized as conditions characterized by excessive threat perception, heightened vigilance, and maladaptive emotional responses. However, contemporary neuroscience increasingly recognizes that anxiety is closely intertwined with the processing and interpretation of bodily signals. Rather than being solely a cognitive or emotional phenomenon, anxiety may emerge from complex interactions among physiological states, interoceptive processing, and predictive regulation [1-5].

Recent reviews and meta-analyses indicate that anxiety is associated with alterations in several dimensions of interoceptive processing, although findings vary according to the interoceptive construct and measurement method examined [19-21]. Individuals with anxiety frequently exhibit increased attention toward bodily sensations, heightened monitoring of physiological states, altered interpretation of interoceptive signals, and greater sensitivity to perceived bodily changes [19-21]. In many cases, anxiety appears to involve not only excessive physiological activation but also alterations in how physiological information is perceived, predicted, and evaluated.

Within predictive coding frameworks, perception arises through continuous interaction between incoming sensory signals and internally generated expectations [1-4]. Under normal conditions, bodily information constrains predictions and contributes to adaptive regulation. However, when sensory information is insufficient, ambiguous, or poorly integrated, prediction errors may become more difficult to resolve, potentially increasing uncertainty regarding the physiological state of the organism.

The free energy principle and active inference models further suggest that biological systems continuously attempt to minimize uncertainty through predictive engagement with their environment [3]. From this perspective, uncertainty is not merely a psychological experience but a biologically relevant state requiring regulation. When uncertainty increases, regulatory systems may respond through heightened vigilance, increased information-seeking behavior, and the enhanced monitoring of potentially relevant signals.

Chronic embodied sensory deprivation could contribute to this process by reducing the quantity and diversity of information available for body-brain calibration. If predictive models rely on continuous sensory updating, the prolonged reduction of embodied input may impair the precision with which physiological states are estimated. Consequently, the nervous system may increasingly rely on prior expectations and internally generated predictions in the absence of sufficient sensory constraints.

Although this proposition remains hypothetical, it offers a plausible mechanism linking embodied sensory impoverishment with anxiety-related phenomena. Specifically, reduced sensory diversity may increase physiological uncertainty, thereby promoting the compensatory attentional monitoring of bodily states.

Within this framework, anxiety may be conceptualized not only as a state of excessive arousal but also as a state of excessive physiological uncertainty. Hypervigilance, the catastrophization of bodily sensations, the intolerance of uncertainty, and the persistent monitoring of internal states may represent attempts to resolve ambiguity regarding the organism’s physiological condition.

Importantly, this model differs from traditional accounts that focus primarily on excessive sensory stimulation. The ESDH framework proposes that dysregulation may emerge from both extremes of the sensory spectrum. Excessive, threatening, or chaotic stimulation may overwhelm regulatory systems, whereas the chronic reduction of embodied sensory information may impair calibration and increase uncertainty. In both cases, maladaptive regulation may result.

This perspective may also help explain why interventions that appear unrelated to cognition can improve anxiety symptoms. Physical exercise, movement-based therapies, yoga, breathing practices, outdoor activities, manual activities, and body-oriented interventions all increase exposure to embodied sensory signals. Although these interventions operate through multiple mechanisms, they share the common feature of increasing sensory interaction between the organism, the body, and the environment.

The concept of sensory enrichment becomes particularly relevant in this context. If embodied sensory deprivation contributes to physiological uncertainty, restoring sensory diversity may enhance predictive calibration and reduce the need for compensatory hypervigilance. Future studies should therefore investigate whether interventions specifically designed to increase embodied sensory exposure can improve interoceptive regulation and reduce anxiety symptoms independently of their established psychological and physiological benefits.

We emphasize that the present model does not propose embodied sensory deprivation as a primary cause of anxiety disorders. Anxiety is a multifactorial condition involving genetic, developmental, environmental, social, and psychological determinants. Rather, ESDH proposes that chronic sensory impoverishment may represent an underrecognized vulnerability factor capable of interacting with established mechanisms of anxiety pathophysiology.

Accordingly, ESDH suggests a theoretical pathway in which the reduction of embodied sensory diversity may impair interoceptive calibration, increase physiological uncertainty, elevate allostatic burden, and contribute to autonomic dysregulation. These processes may, in turn, promote hypervigilance and anxiety-related symptomatology.

This proposed pathway remains speculative and requires empirical validation. However, it provides a coherent framework through which contemporary findings in interoception, predictive neuroscience, and anxiety research may be integrated into a unified explanatory model. The proposed relationship is unlikely to be unidirectional. Anxiety, hypervigilance, avoidance, physical inactivity, and social withdrawal may themselves further reduce embodied sensory exposure, thereby creating reciprocal or multidirectional feedback loops that could maintain or amplify dysregulation over time.

Relevance to psychosomatic symptoms and somatic symptom amplification

Psychosomatic symptoms represent a complex interface between physiological processes, emotional regulation, cognitive appraisal, and bodily perception. Although the term “psychosomatic” has historically generated considerable debate, contemporary models increasingly recognize that symptom generation and symptom perception emerge from dynamic interactions between the brain and the body, rather than from a simple distinction between psychological and organic causes. Many functional and psychosomatic conditions are characterized by alterations in the perception, interpretation, and regulation of bodily signals. In somatic symptom disorder, illness anxiety disorder, and several functional syndromes, symptom burden may not be fully accounted for by identifiable structural abnormalities, despite genuine distress and functional impairment [22]. These observations have contributed to the development of modern body-brain interaction models emphasizing the importance of interoceptive processing and predictive regulation in symptom perception.

Recent evidence supports the relevance of interoception in somatic symptom disorders and related functional conditions. A systematic review and meta-analysis by Wolters et al. demonstrated that somatic symptom disorder, illness anxiety disorder, and several functional syndromes are associated with alterations in interoceptive accuracy and response bias [22]. These findings suggest that disturbances in the processing of bodily signals may contribute to symptom amplification and altered symptom perception. From a predictive coding perspective, symptom perception does not arise solely from incoming sensory signals. Rather, symptoms emerge through continuous interaction between sensory information, prior expectations, attentional processes, and contextual interpretation [1-4]. Consequently, the experience of bodily symptoms may be influenced not only by peripheral physiological processes but also by the quality of body-brain communication and predictive regulation. The Embodied Sensory Deprivation Hypothesis extends this framework by proposing that chronic reduction in embodied sensory diversity may contribute to a progressive weakening of body-brain calibration mechanisms.

We hypothesize that when the nervous system is exposed to a reduced range of meaningful sensory experiences, its capacity to model and interpret physiological states may become less precise. Under these conditions, ordinary bodily fluctuations may be more likely to generate uncertainty, attract attention, and be interpreted as potentially significant or threatening. Importantly, ESDH does not propose that psychosomatic symptoms are caused by sensory deprivation. Such a claim would oversimplify the multifactorial nature of these conditions and would not be supported by current evidence. Rather, the hypothesis frames embodied sensory impoverishment as one potential factor capable of influencing symptom perception, amplification, and persistence. Within this framework, sensory malnutrition may contribute to somatic symptom amplification through several potential mechanisms. Reduced sensory diversity may limit opportunities for the continuous calibration of predictive body models, thereby increasing uncertainty regarding internal physiological states. Insufficient embodied input may also reduce the efficiency with which predictive models are updated in response to changing physiological conditions. Increased uncertainty may promote greater attentional allocation toward bodily sensations, enhancing symptom awareness and salience. Persistent uncertainty may require greater regulatory effort, potentially contributing to elevated allostatic burden, autonomic dysregulation, and chronic stress-related physiological responses. Finally, when predictive calibration is impaired, ordinary physiological sensations may be more likely to be perceived as unusual, significant, or threatening. Taken together, these mechanisms provide a theoretical pathway through which embodied sensory deprivation could contribute to somatic symptom amplification without requiring structural pathology or direct sensory dysfunction.

An important implication of this model is that psychosomatic symptoms may not merely reflect excessive symptom focus but could also reflect insufficient exposure to normalizing embodied sensory experiences. In other words, symptom amplification may emerge not only because individuals attend excessively to bodily signals but also because the nervous system receives too little diverse information with which to contextualize and interpret those signals. This perspective generates the hypothesis that interventions increasing movement and embodied engagement might influence symptom perception through mechanisms extending beyond the specific physiological system targeted. This possibility remains to be tested directly. Although these interventions undoubtedly operate through multiple mechanisms, they all increase embodied sensory engagement and may therefore contribute to the restoration of body-brain calibration. We emphasize that this proposition remains speculative and requires empirical investigation. Nevertheless, ESDH provides a coherent framework through which contemporary findings in psychosomatic medicine, interoception, and predictive neuroscience may be integrated into a broader model of embodied regulation. Accordingly, psychosomatic symptoms may represent one possible downstream manifestation of chronic sensory malnutrition when combined with individual vulnerability factors, environmental stressors, and altered interoceptive processing.

Relevance to Disorders of Gut-Brain Interaction (DGBI)

Disorders of Gut-Brain Interaction (DGBI) provide a clinically relevant context for exploring the implications of the Embodied Sensory Deprivation Hypothesis. Formerly referred to as functional gastrointestinal disorders, DGBI are now understood as complex conditions arising from dynamic interactions among gastrointestinal physiology, central nervous system processing, autonomic regulation, immune function, microbial ecology, and behavioral factors [23,24].

The Rome IV framework emphasized that symptoms such as abdominal pain, bloating, altered bowel habits, and visceral discomfort cannot be adequately explained through a purely structural model of disease. Instead, DGBI are increasingly conceptualized as disorders involving altered communication between the gastrointestinal tract and the central nervous system [23,24].

Among the mechanisms implicated in DGBI, altered visceral sensitivity and the abnormal central processing of internal bodily signals are considered particularly relevant. Patients with DGBI may exhibit heightened attention to gastrointestinal sensations, gastrointestinal symptom-specific anxiety, visceral hypervigilance, and amplified responses to physiological visceral stimuli [25,26].

Recent evidence suggests that altered interoceptive processing may contribute to symptom perception in DGBI. Karaivazoglou et al. reviewed evidence indicating that patients with functional gastrointestinal disorders may show alterations in the perception, interpretation, and central integration of visceral afferent signals [25]. These findings are consistent with broader models proposing that symptom generation in DGBI depends not only on peripheral gastrointestinal events but also on how those events are processed within the central nervous system.

From the perspective of predictive coding, gastrointestinal sensations are continuously interpreted in the context of prior expectations and ongoing predictions regarding bodily state [1-4]. Visceral sensations are therefore not passively perceived but actively constructed through interactions between afferent signals and predictive regulatory mechanisms. Disturbances in this process may contribute to visceral hypersensitivity, symptom amplification, and maladaptive behavioral responses [25,26].

ESDH extends this framework by proposing that disturbances in gut-brain communication may occur within a broader context of impaired body-brain calibration. We hypothesize that chronic reduction in embodied sensory diversity may diminish the richness of information available for predictive regulation, thereby increasing physiological uncertainty and altering the interpretation of visceral signals.

Within this model, gastrointestinal sensations may become disproportionately salient because they represent one of the most continuously available sources of internal bodily information. When overall embodied sensory input is reduced, the nervous system may increasingly rely on visceral signals to infer physiological state. Under such conditions, ordinary gastrointestinal sensations may acquire greater attentional and emotional significance.

Although this proposition remains speculative, it may help explain several commonly observed features of DGBI. Increased attention toward gastrointestinal sensations may emerge as a compensatory response to uncertainty regarding bodily state, contributing to visceral hypervigilance. Ordinary visceral sensations may be interpreted as more intense or clinically significant when predictive calibration is impaired, thereby promoting symptom amplification. Heightened physiological uncertainty may simultaneously increase anxiety and gastrointestinal symptom perception, reinforcing anxiety-gut symptom coupling. In addition, reduced sensory diversity may contribute to altered autonomic balance, potentially influencing gastrointestinal motility, secretion, sensitivity, and symptom generation. Once established, maladaptive predictive models may also reinforce symptom persistence even when peripheral physiological abnormalities are minimal.

Importantly, ESDH does not seek to replace existing models of DGBI. The roles of microbiota composition, intestinal permeability, immune activation, motility disturbances, dietary factors, and psychosocial stressors remain well-established and should continue to be regarded as central components of DGBI pathophysiology [23-25]. Rather, the present hypothesis proposes that embodied sensory impoverishment may represent an additional layer of vulnerability capable of interacting with these established mechanisms. This relationship should be understood as bidirectional rather than linear. Sensory malnutrition may contribute to impaired visceral predictive calibration, increased physiological uncertainty, and visceral hypervigilance; however, established DGBI symptoms may also promote behaviors that further reduce embodied sensory exposure. Patients with DGBI may restrict activities, social participation, travel, eating situations, or other contexts associated with anticipated symptom exacerbation [26]. Within the ESDH framework, we further hypothesize that such avoidance could indirectly reduce exposure to movement, social contact, environmental variability, and other embodied sensory experiences.

Such avoidance may initially function as an adaptive coping strategy aimed at preventing symptom flares. Over time, however, it may reduce exposure to proprioceptive, interoceptive, tactile, environmental, and social sensory inputs that could otherwise support body-brain recalibration. Psychological and central-processing models of DGBI support reciprocal interactions among visceral symptoms, threat-related expectations, attention, and avoidance behavior [26]. Extending these models, we propose that reduced embodied sensory exposure could become an additional maintaining component within this self-reinforcing cycle. If repeatedly reinforced over time, these patterns of avoidance and reduced embodied engagement may become increasingly habitual, thereby stabilizing sensory impoverishment and making the cycle more difficult to reverse. This possibility should be understood primarily at the behavioral and learning level, as direct evidence linking ESDH to specific synaptic plasticity mechanisms is currently lacking.

This perspective may also help explain why interventions not directly targeting the gastrointestinal tract often produce clinically meaningful benefits in patients with DGBI. Several non-pharmacological approaches used in selected patients with DGBI, including physical activity, yoga, breathing-based practices, and mindfulness-oriented interventions, involve increased engagement with bodily sensations and movement. Their clinical effects are likely multifactorial, and the possibility that sensory enrichment contributes to these effects remains hypothetical.

Within the ESDH framework, DGBI may therefore be conceptualized not only as disorders of gut-brain communication but also as potential manifestations of broader disturbances in embodied regulation. Future studies should investigate whether markers of sensory malnutrition correlate with symptom severity, visceral hypersensitivity, autonomic dysfunction, and quality of life in patients with DGBI.

If confirmed, this perspective could open new avenues for integrative treatment strategies aimed not only at modifying gastrointestinal physiology but also at restoring the diversity and richness of embodied sensory experience.

Sedentary behavior, physical inactivity, and sensory malnutrition

Among the various components of embodied sensory deprivation, physical inactivity and sedentary behavior may represent some of the most pervasive and measurable manifestations of sensory impoverishment in contemporary societies. Sedentary behavior has been extensively investigated in relation to cardiometabolic disease, cardiovascular outcomes, diabetes, and mortality [27]. Its potential implications for embodied sensory regulation, however, have received comparatively little direct investigation.

Sedentary behavior is defined as any waking behavior characterized by an energy expenditure of ≤1.5 metabolic equivalents while in a sitting, reclining, or lying posture [28]. Technological development, motorized transportation, and increasingly sedentary occupational and recreational activities have contributed to high levels of sedentary time in contemporary populations [28]. Sedentary behavior commonly occurs across occupational, transport, and leisure domains [29,30].

A growing body of evidence suggests that sedentary behavior is associated with adverse mental health outcomes. Systematic reviews and meta-analyses have demonstrated positive associations between sedentary behavior and anxiety symptoms across different age groups and populations [31,32]. Conversely, higher levels of physical activity are associated with a lower risk of anxiety and improved psychological well-being [33].

Several biological, psychological, and social mechanisms have been proposed to explain the association between physical activity and anxiety; however, their relative contributions remain incompletely understood. While these explanations remain highly relevant, the ESDH framework proposes an additional perspective: physical inactivity may also be understood as a reduction in embodied sensory exposure.

Every episode of movement generates a complex constellation of sensory signals, including proprioceptive feedback from muscles and joints, vestibular information related to spatial orientation, mechanosensory stimulation from contact with surfaces, cardiorespiratory fluctuations, thermoregulatory adjustments, and dynamic interoceptive signals arising from physiological adaptation to effort.

Consequently, physical activity represents not only a means of energy expenditure but also a continuous source of sensory nutrition. Through movement, the nervous system receives rich streams of information regarding posture, balance, effort, environmental interaction, and physiological state. When movement is substantially reduced, many of these signals decrease in frequency, intensity, and variability. Individuals may continue to receive visual and cognitive stimulation yet experience a marked reduction in proprioceptive, vestibular, mechanosensory, and cardiorespiratory inputs. From the perspective of ESDH, this reduction may contribute to a broader state of sensory malnutrition.

Sedentary behavior may therefore represent a particularly informative model through which to investigate the broader hypothesis. Unlike more difficult-to-measure variables such as social touch or environmental complexity, physical inactivity can be objectively quantified through accelerometry, wearable technologies, and behavioral assessment tools. This provides a practical entry point for future empirical studies.

Within the proposed framework, physical inactivity may contribute to embodied sensory deprivation through several pathways. Limited movement reduces exposure to changes in posture, muscular tension, balance demands, and spatial orientation, thereby decreasing proprioceptive diversity. It also reduces physiological variability, because physical activity normally generates fluctuations in heart rate, respiration, temperature, and metabolic state. Inactive lifestyles may further limit environmental interaction, sensory novelty, and interoceptive challenge, as individuals are exposed to fewer opportunities for exploration, adaptation to variable physical contexts, and the calibration of internal physiological signals through effort.

We hypothesize that these factors may collectively contribute to diminished body-brain calibration and increased physiological uncertainty. Although current evidence does not directly demonstrate such a mechanism, the association between sedentary behavior and anxiety-related outcomes is consistent with the possibility that movement serves important regulatory functions extending beyond its metabolic effects [31-33].

An additional implication of this perspective concerns the interpretation of exercise interventions. Physical activity is often conceptualized as a treatment for anxiety, depression, and stress through biochemical mechanisms such as endorphin release, neurotrophic signaling, and anti-inflammatory effects. While these mechanisms undoubtedly contribute, ESDH suggests that exercise may also function as a form of sensory enrichment.

Movement restores exposure to proprioceptive, vestibular, mechanosensory, cardiorespiratory, and interoceptive signals that may have become diminished during prolonged periods of inactivity. Consequently, part of the therapeutic effect of exercise may derive from the restoration of embodied sensory diversity and the improved calibration of body-brain regulatory systems.

This interpretation remains speculative but generates several testable predictions. Interventions emphasizing sensory richness, environmental variability, and complex movement patterns may theoretically produce greater benefits than interventions focused exclusively on energy expenditure. Similarly, activities involving natural environments, balance challenges, manual engagement, and multisensory interaction may provide additional regulatory benefits through the enhancement of sensory nutrition.

Within the ESDH framework, sedentary behavior therefore represents more than a risk factor for metabolic disease. It may also serve as a measurable indicator of embodied sensory deprivation and a potential contributor to sensory malnutrition. Future research should determine whether objective measures of physical inactivity correlate with markers of interoceptive dysregulation, autonomic instability, anxiety severity, and symptom burden across clinical populations.

From hypothesis to clinical syndrome: The proposed construct of Embodied Input Deficiency Syndrome (EIDS)

At the present stage, the Embodied Sensory Deprivation Hypothesis (ESDH) should be regarded as a theoretical framework intended to generate testable predictions regarding the relationship between sensory impoverishment, body-brain regulation, and symptom development.

The available literature supports the relevance of interoception, predictive processing, allostatic regulation, affective touch, physical activity, and gut-brain communication in human health and disease [1-9,18-22]. However, direct evidence demonstrating that the chronic reduction of embodied sensory diversity constitutes a unified clinical phenomenon remains limited.

Accordingly, ESDH should not currently be interpreted as a diagnostic model. Rather, it should be viewed as a conceptual framework capable of integrating observations that are presently dispersed across multiple disciplines, including neuroscience, psychosomatic medicine, lifestyle medicine, behavioral medicine, gastroenterology, and evolutionary medicine.

Nevertheless, if future research identifies a reproducible phenotype characterized by chronic embodied sensory impoverishment, altered interoceptive regulation, and a consistent pattern of physiological and psychological manifestations, it may become appropriate to define a formal clinical research construct. Such a phenotype could provisionally be termed Embodied Input Deficiency Syndrome (EIDS).

Embodied Input Deficiency Syndrome (EIDS)

EIDS would represent the putative clinical expression of chronic embodied sensory deprivation.

Importantly, EIDS would not be defined by the absence of a single sensory modality. Rather, it would describe a multidimensional condition characterized by insufficient exposure to the diversity of embodied sensory experiences required for optimal nervous system regulation.

Within this framework, EIDS may occupy a position analogous to other deficiency-based models in medicine. Just as nutritional deficiencies arise not necessarily from the complete absence of nutrients but from chronic inadequacy relative to physiological requirements, EIDS would represent a state in which embodied sensory exposure falls below the threshold necessary to maintain efficient body-brain calibration.

The syndrome would therefore be conceptualized not as a psychiatric disorder, neurological disease, or gastrointestinal condition per se but as a transdiagnostic vulnerability state capable of influencing multiple physiological systems.

Proposed Preliminary Research Criteria for EIDS

The following preliminary criteria are proposed exclusively as a research framework and should not be interpreted as diagnostic criteria. They are summarized in Table 3.

**Table 3:** Proposed preliminary research criteria for Embodied Input Deficiency Syndrome (EIDS). Original conceptual framework proposed by the authors. HRV, heart rate variability; DGBI, Disorders of Gut-Brain Interaction

Criterion	Core feature	Examples
A	Chronic embodied sensory impoverishment	Physical inactivity, reduced touch, reduced environmental variability, and limited manual activity
B	Altered body-brain regulation	Interoceptive dysregulation, autonomic instability, reduced HRV, and somatic hypervigilance
C	Clinical manifestations	Anxiety, psychosomatic symptoms, DGBI, and functional syndromes
D	Responsiveness to sensory enrichment	Improvement after movement, outdoor exposure, proprioceptive training, and body-oriented therapies

EIDS as a Transdiagnostic Vulnerability Model

A central feature of the proposed construct is its transdiagnostic nature. EIDS does not imply that sensory deprivation directly causes specific diseases. Rather, chronic embodied sensory impoverishment is proposed as a potential vulnerability factor that may interact with genetic, developmental, psychological, microbial, immune, lifestyle, and social determinants of health. Under this model, EIDS may function as a predisposing, disease-modifying, perpetuating, or resilience-reducing factor. Different individuals may therefore express different clinical manifestations despite sharing a similar underlying state of sensory malnutrition. For example, one individual may predominantly develop anxiety-related symptoms, another may exhibit somatic symptom amplification, another may develop DGBI manifestations, and others may experience overlapping presentations.

This perspective is consistent with contemporary transdiagnostic models of psychopathology and psychosomatic medicine, which increasingly emphasize shared mechanisms rather than strictly disease-specific pathways.

Proposed Developmental Sequence

The ESDH framework suggests a theoretical developmental sequence in which modern sensory environments reduce exposure to movement, affective touch, and environmental variability. This reduction may contribute to sensory malnutrition and embodied sensory deprivation, which in turn may impair interoceptive calibration, increase physiological uncertainty, elevate allostatic burden, and promote autonomic dysregulation. These processes may increase vulnerability to anxiety-related symptoms, somatic hypervigilance, psychosomatic symptoms, and Disorders of Gut-Brain Interaction, potentially contributing to the future clinical expression of EIDS.

This sequence should be regarded as a theoretical model rather than an established causal pathway.

Implications for Clinical Research

The introduction of EIDS as a research construct may provide several advantages. First, it offers a framework for investigating whether modern sensory environments influence health through mechanisms extending beyond traditional psychological and metabolic pathways. Second, it encourages the development of tools capable of quantifying sensory exposure across multiple domains. Third, it generates testable hypotheses regarding the therapeutic potential of sensory enrichment interventions. Finally, it provides a common conceptual language through which observations from neuroscience, psychiatry, psychosomatic medicine, gastroenterology, rehabilitation medicine, and lifestyle medicine may be integrated.

At present, the existence of EIDS remains hypothetical. Nevertheless, the construct may provide a useful platform for future observational, mechanistic, and interventional studies aimed at understanding how embodied sensory experience contributes to human health and disease.

Research agenda and testable predictions

For a theoretical framework to contribute meaningfully to scientific progress, it must generate empirically testable predictions. The Embodied Sensory Deprivation Hypothesis is intended not as a definitive explanation of anxiety, psychosomatic symptoms, or Disorders of Gut-Brain Interaction but as a model capable of guiding future observational, mechanistic, and interventional research.

The central proposition of ESDH is that chronic reduction in embodied sensory diversity may contribute to impaired body-brain calibration, increased physiological uncertainty, and vulnerability to conditions characterized by altered interoceptive regulation. This proposition generates a series of specific and potentially falsifiable predictions, summarized in Table 4.

**Table 4:** Testable predictions generated by the Embodied Sensory Deprivation Hypothesis. Original hypotheses and testable predictions proposed by the authors, informed by literature on interoception, anxiety, DGBI, and physical activity [1-5,18-26,28,31-33]. HRV, heart rate variability; DGBI, Disorders of Gut-Brain Interaction

Prediction	Expected association	Possible measures
Sensory malnutrition and interoception	Lower sensory diversity associated with altered interoceptive function	Interoceptive accuracy, awareness, and confidence
Sensory malnutrition and anxiety	Lower embodied exposure associated with higher anxiety	Anxiety scales, health anxiety, and intolerance of uncertainty
DGBI and sensory impoverishment	Patients with DGBI may show reduced sensory exposure	Activity, touch, nature exposure, and visceral hypersensitivity
Sedentary behavior as proxy	Greater sedentary time associated with dysregulation	Accelerometry, wearable devices, and movement variability
Sensory enrichment	Multimodal enrichment improves symptoms	HRV, anxiety, DGBI severity, and quality of life
Sensory diversity and resilience	Higher diversity associated with better regulation	HRV, stress resilience, and symptom burden

Development of a Sensory Exposure Index

A major objective for future research should be the development of standardized instruments capable of quantifying embodied sensory exposure across multiple domains. Such an instrument could be provisionally termed the Embodied Sensory Exposure Index (ESEI). The ESEI should evaluate not only the quantity of sensory exposure but also its diversity, variability, and biological relevance. Potential domains may include movement, proprioception, environmental variability, affective touch, social contact, manual activity, thermal exposure, and interaction with natural environments. Such a tool would provide an operational framework for investigating sensory malnutrition and EIDS across populations. It would also allow researchers to examine whether specific sensory profiles are associated with interoceptive dysregulation, autonomic instability, anxiety-related symptoms, DGBI severity, or responsiveness to sensory enrichment interventions.

A Proposed Research Pathway

The development of ESDH and EIDS may proceed through four sequential phases, as summarized in Table 5.

**Table 5:** Proposed research pathway for ESDH and EIDS. Original research pathway proposed by the authors. HRV, heart rate variability; DGBI, Disorders of Gut-Brain Interaction; ESDH, Embodied Sensory Deprivation Hypothesis; EIDS, Embodied Input Deficiency Syndrome

Phase	Objective	Main focus
Phase 1	Observational studies	Identify associations between sensory exposure and clinical outcomes: sensory exposure, anxiety, DGBI, symptom burden, and quality of life
Phase 2	Mechanistic studies	Evaluate biological and neurophysiological mechanisms: interoception, HRV, autonomic regulation, and predictive processing
Phase 3	Intervention studies	Assess sensory enrichment strategies: movement, touch, nature exposure, and multimodal enrichment
Phase 4	Clinical validation	Determine whether EIDS represents a reproducible construct: phenotype definition, diagnostic boundaries, and treatment responsiveness

At present, ESDH remains a hypothesis. However, by generating specific and testable predictions, the framework satisfies a central requirement of scientific theory: the ability to be challenged, refined, or falsified through empirical investigation.

Clinical implications

If the Embodied Sensory Deprivation Hypothesis proves valid, its implications may extend beyond theoretical neuroscience and contribute to the development of novel preventive and therapeutic approaches. The primary clinical value of ESDH lies not in the identification of a new disease category but in the introduction of a previously underrecognized dimension of health: the quality and diversity of embodied sensory experience.

Contemporary clinical practice routinely evaluates nutrition, physical activity, sleep quality, psychological stress, and social support. However, relatively little attention is devoted to the assessment of embodied sensory exposure as a determinant of physiological regulation. From the perspective of ESDH, this omission may represent a significant gap in current models of health and disease.

The present framework suggests that clinicians may benefit from considering not only what patients eat, think, or feel but also how they physically interact with their bodies and environments. Questions concerning movement, sensory variability, social touch, environmental engagement, and bodily experience may provide valuable information regarding the integrity of body-brain regulation.

Importantly, ESDH does not imply that sensory deprivation should replace established risk factors or therapeutic targets. Rather, embodied sensory exposure should be viewed as an additional dimension interacting with known biological, psychological, and social determinants of health.

The Concept of Sensory Enrichment

Within the ESDH framework, the logical counterpart of sensory malnutrition is sensory enrichment.

Sensory enrichment may be defined as the intentional restoration of meaningful embodied sensory experiences capable of increasing the diversity and richness of body-brain interactions.

The objective of sensory enrichment is not the indiscriminate increase of stimulation. Excessive or chaotic sensory exposure may itself become dysregulating. Rather, sensory enrichment seeks to restore biologically relevant forms of sensory experience that support adaptive regulation and predictive calibration.

From this perspective, sensory enrichment may represent a process analogous to nutritional rehabilitation. Just as dietary interventions aim to restore adequate nutrient intake, sensory enrichment interventions aim to restore adequate embodied sensory exposure.

Potential Domains of Sensory Enrichment

Several forms of intervention may contribute to increased sensory nutrition by restoring biologically meaningful embodied inputs across multiple domains.

Movement-based enrichment represents one of the most accessible and biologically relevant strategies. Activities such as walking, hiking, resistance training, balance exercises, climbing, dance, martial arts, and sports involving complex movement patterns may increase proprioceptive, vestibular, mechanosensory, and interoceptive stimulation while simultaneously promoting physiological variability.

Environmental enrichment may also play an important role. Natural environments provide more diverse sensory experiences than standardized indoor settings, offering exposure to green spaces, outdoor movement, varied terrains, seasonal conditions, and ecological complexity. Such environments may combine sensory richness, unpredictability, and biological relevance in ways that support adaptive body-brain regulation.

Social and affective enrichment represents another relevant domain. Human nervous systems evolved within contexts characterized by frequent interpersonal interaction, physical presence, and social contact. Meaningful sources of social sensory enrichment may include affectionate touch, cooperative activities, group exercise, family interaction, and other forms of embodied social engagement. Although not all forms of social contact are beneficial, safe and meaningful interpersonal connection may represent an important component of sensory nutrition.

Manual and exploratory enrichment may further contribute to embodied sensory diversity. Activities such as gardening, craftsmanship, manual work, playing musical instruments, artistic activities, and practical skill development require active interaction with the physical world and may increase tactile, proprioceptive, and visuomotor engagement.

Thermal and environmental variability may represent an additional component of sensory enrichment. Historically, humans experienced substantial thermal variability throughout daily life. Controlled exposure to outdoor conditions across seasons, moderate cold, moderate heat, and variable environmental contexts may enrich thermosensory signaling and support physiological adaptability.

Together, these domains suggest that sensory enrichment should not be understood as a single intervention but as a multidimensional strategy aimed at restoring the diversity, variability, and biological relevance of embodied sensory experience.

A Multidimensional Approach

A central implication of ESDH is that no single intervention is likely to fully address sensory malnutrition. Because sensory deprivation is proposed to be cumulative and multidimensional, effective sensory enrichment may require simultaneous engagement across multiple sensory domains.

For example, a simple walk in a natural environment with a companion may combine locomotion, proprioceptive stimulation, environmental variability, social interaction, visual complexity, and thermosensory exposure. From the perspective of ESDH, such activities may provide richer sensory nutrition than interventions targeting a single domain in isolation.

This multidimensional view also suggests that interventions characterized by ecological complexity, bodily engagement, and contextual variability may be particularly relevant for future research.

Potential Applications in Clinical Populations

Although empirical validation is still required, sensory enrichment strategies may eventually prove relevant in clinical populations characterized by altered body-brain regulation. In anxiety-related conditions, sensory enrichment may help improve predictive calibration and reduce physiological uncertainty. In psychosomatic and functional symptoms, increased embodied sensory diversity may support more adaptive symptom interpretation and reduce somatic amplification. In Disorders of Gut-Brain Interaction, enhanced body-brain regulation may contribute to improved autonomic balance and reduced visceral hypervigilance.

Sensory enrichment may also be relevant in stress-related conditions, where it could support allostatic flexibility and resilience, and in lifestyle-related disorders, where it may complement established approaches focused on diet, physical activity, and sleep. These potential applications remain hypothetical, but they provide a rationale for future observational and interventional studies evaluating sensory enrichment as a transdiagnostic strategy.

Discussion

The Embodied Sensory Deprivation Hypothesis (ESDH) proposes an integrative model linking interoception, predictive neuroscience, active inference, allostasis, evolutionary medicine, and Disorders of Gut-Brain Interaction within a unified framework of body-brain regulation. Its central premise is that modern environments may progressively reduce the diversity and variability of biologically meaningful sensory experiences. Although contemporary lifestyles have improved safety, comfort, and longevity, they may also have diminished exposure to sensory conditions that historically contributed to the calibration of neural regulatory systems. ESDH does not seek to replace existing explanations for anxiety, psychosomatic symptoms, or DGBI. Rather, it adds a complementary dimension: the possibility that embodied sensory experience itself functions as a regulatory resource. Several observations can be reconsidered in the light of this model. Evidence supports beneficial health associations for specific forms of embodied engagement, including affective touch and physical activity [9,33]. Whether these heterogeneous experiences share a common effect through the restoration of embodied sensory diversity remains an original hypothesis of the ESDH framework.

These effects are usually attributed to mechanisms such as neuroplasticity, autonomic regulation, social support, inflammation modulation, and behavioral activation. ESDH raises the possibility that these apparently heterogeneous interventions may also share a common feature: the restoration of embodied sensory diversity. In this view, movement is not merely exercise, touch is not merely social contact, and environmental exposure is not merely recreation. Each may represent a form of sensory nutrition capable of supporting predictive calibration and physiological regulation. A key element of the model is physiological uncertainty. Predictive frameworks emphasize that biological systems continuously attempt to minimize uncertainty through active engagement with sensory information.

If embodied sensory experiences contribute to this process, chronic sensory impoverishment may theoretically increase uncertainty regarding bodily state, thereby promoting compensatory hypervigilance and maladaptive regulation. This perspective may help explain why anxiety, psychosomatic symptoms, and DGBI frequently coexist. Rather than representing entirely separate conditions, they may share disturbances in body-brain communication, interoceptive processing, and predictive regulation. Importantly, ESDH should not be interpreted as a reductionist explanation.

The hypothesis does not suggest that sensory deprivation alone causes disease. Instead, sensory malnutrition is proposed as a potential vulnerability factor interacting with genetics, development, trauma, lifestyle, microbiota, immune function, social context, and psychological processes. A further strength of the model is its falsifiability. ESDH generates specific predictions regarding sensory exposure, interoceptive regulation, autonomic function, and clinical outcomes. These predictions can be tested through observational studies, physiological assessments, and controlled interventions. Finally, ESDH does not equate sensory malnutrition with physical inactivity, social isolation, or reduced touch alone. Sensory malnutrition is proposed as an emergent multidimensional construct arising from the cumulative reduction of multiple embodied sensory domains. Ultimately, the value of ESDH will depend not on its conceptual appeal but on its ability to generate reproducible empirical findings. The present work should therefore be regarded as a starting point for scientific investigation rather than a definitive explanatory model.

Limitations

Several limitations should be acknowledged. First, ESDH remains a theoretical construct. Although its individual components are supported by existing literature, direct evidence demonstrating a unified syndrome of embodied sensory deprivation is currently lacking. Second, many of the proposed mechanisms remain speculative. While contemporary models support important roles for interoception, predictive processing, and allostatic regulation, the specific relationship between sensory malnutrition and clinical symptom development has not yet been experimentally established. Third, sensory nutrition has not yet been operationally defined. At present, no validated instrument exists to quantify embodied sensory exposure across multiple domains. The development of such measures is therefore a necessary prerequisite for future empirical investigation. Fourth, substantial interindividual heterogeneity is likely. The quantity and type of sensory experiences required for optimal regulation may vary according to age, personality, cultural context, physical health, developmental history, and environmental circumstances. Fifth, although the revised framework explicitly distinguishes embodied sensory deficiency from modern exteroceptive and informational overload, the interaction between these two dimensions remains insufficiently characterized. Future models should more precisely investigate how digital, visual, and cognitive overload interacts with reduced proprioceptive, interoceptive, tactile, thermosensory, and environmental input within a broader sensory ecology. Finally, caution is warranted in interpreting EIDS as a potential diagnostic entity. At present, Embodied Input Deficiency Syndrome should be considered solely as a proposed research construct intended to facilitate scientific investigation. Substantial evidence would be required before any clinical application could be justified.

## Conclusions

The Embodied Sensory Deprivation Hypothesis (ESDH) proposes that chronic reduction in the quantity, diversity, and variability of embodied sensory experiences may represent an underrecognized influence on body-brain regulation. Drawing on interoception, predictive coding, active inference, allostasis, and evolutionary mismatch, ESDH introduces the concept of sensory malnutrition as insufficient exposure to biologically meaningful sensory experiences required for optimal neurophysiological functioning. Within this framework, embodied sensory deprivation is proposed as a potential contributor to impaired interoceptive calibration, increased physiological uncertainty, elevated allostatic burden, and vulnerability to conditions characterized by altered body-brain communication. Anxiety-related symptoms, psychosomatic manifestations, and Disorders of Gut-Brain Interaction represent particularly relevant contexts in which these mechanisms may operate. The hypothesis further suggests that, if future research identifies a reproducible phenotype characterized by chronic sensory impoverishment, altered interoceptive regulation, and responsiveness to sensory enrichment interventions, a formal clinical construct provisionally termed Embodied Input Deficiency Syndrome (EIDS) may eventually be justified. At present, both ESDH and EIDS should be regarded as research frameworks rather than established clinical entities. Their scientific value will depend on empirical validation. Nevertheless, ESDH offers a novel framework for understanding modern environmental conditions, embodied sensory experience, and body-brain regulation as interconnected dimensions of health. By integrating findings from neuroscience, psychosomatic medicine, lifestyle medicine, evolutionary biology, and gut-brain interaction research, it provides a foundation for future investigation into the role of sensory nutrition in human health and disease.

## References

[1] Barrett LF, Simmons WK (2015). Interoceptive predictions in the brain. Nat Rev Neurosci.

[2] Barrett LF, Quigley KS, Hamilton P (2016). An active inference theory of allostasis and interoception in depression. Philos Trans R Soc Lond B Biol Sci.

[3] Friston K (2010). The free-energy principle: a unified brain theory?. Nat Rev Neurosci.

[4] Owens AP, Allen M, Ondobaka S, Friston KJ (2018). Interoceptive inference: from computational neuroscience to clinic. Neurosci Biobehav Rev.

[5] Chen WG, Schloesser D, Arensdorf AM (2021). The emerging science of interoception: sensing, integrating, interpreting, and regulating signals within the self. Trends Neurosci.

[6] Gluckman PD, Hanson MA, Low FM (2019). Evolutionary and developmental mismatches are consequences of adaptive developmental plasticity in humans and have implications for later disease risk. Philos Trans R Soc Lond B Biol Sci.

[7] Gurven MD, Lieberman DE (2020). WEIRD bodies: mismatch, medicine and missing diversity. Evol Hum Behav.

[8] McGlone F, Wessberg J, Olausson H (2014). Discriminative and affective touch: sensing and feeling. Neuron.

[9] Packheiser J, Hartmann H, Fredriksen K, Gazzola V, Keysers C, Michon F (2024). A systematic review and multivariate meta-analysis of the physical and mental health benefits of touch interventions. Nat Hum Behav.

[10] Spitz RA (1945). Hospitalism; an inquiry into the genesis of psychiatric conditions in early childhood. Psychoanal Study Child.

[11] Spitz RA, Cobliner WG, Freud A (1965). The first year of life : a psychoanalytic study of normal and deviant development of object relations. The first year of life : a psychoanalytic study of normal and deviant development of object relations.

[12] Boundy EO, Dastjerdi R, Spiegelman D (2016). Kangaroo mother care and neonatal outcomes: a meta-analysis. Pediatrics.

[13] Conde-Agudelo A, Diaz-Rossello JL, Belizan JM (2003). Kangaroo mother care to reduce morbidity and mortality in low birthweight infants. Cochrane Database Syst Rev.

[14] Arya S, Naburi H, Kawaza K (2021). Immediate “kangaroo mother care” and survival of infants with low birth weight. N Engl J Med.

[15] Sivanandan S, Sankar MJ (2023). Kangaroo mother care for preterm or low birth weight infants: a systematic review and meta-analysis. BMJ Glob Health.

[16] Harlow HF (1958). The nature of love. Am Psychol.

[17] von Mohr M, Kirsch LP, Fotopoulou A (2021). Social touch deprivation during COVID-19: effects on psychological wellbeing and craving interpersonal touch. R Soc Open Sci.

[18] Wallman-Jones A, Perakakis P, Tsakiris M, Schmidt M (2021). Physical activity and interoceptive processing: theoretical considerations for future research. Int J Psychophysiol.

[19] Clemente R, Murphy A, Murphy J (2024). The relationship between self-reported interoception and anxiety: a systematic review and meta-analysis. Neurosci Biobehav Rev.

[20] Adams KL, Edwards A, Peart C, Ellett L, Mendes I, Bird G, Murphy J (2022). The association between anxiety and cardiac interoceptive accuracy: a systematic review and meta-analysis. Neurosci Biobehav Rev.

[21] Jenkinson PM, Fotopoulou A, Ibañez A, Rossell S (2024). Interoception in anxiety, depression, and psychosis: a review. EClinicalMedicine.

[22] Wolters C, Gerlach AL, Pohl A (2022). Interoceptive accuracy and bias in somatic symptom disorder, illness anxiety disorder, and functional syndromes: a systematic review and meta-analysis. PLoS One.

[23] Drossman DA, Hasler WL (2016). Rome IV-functional GI disorders: disorders of gut-brain interaction. Gastroenterology.

[24] Drossman DA (2016). Functional gastrointestinal disorders: History, pathophysiology, clinical features and Rome IV. Gastroenterology.

[25] Karaivazoglou K, Aggeletopoulou I, Triantos C (2024). Interoceptive processing in functional gastrointestinal disorders. Int J Mol Sci.

[26] Van Oudenhove L, Aziz Q (2009). Recent insights on central processing and psychological processes in functional gastrointestinal disorders. Dig Liver Dis.

[27] Patterson R, McNamara E, Tainio M (2018). Sedentary behaviour and risk of all-cause, cardiovascular and cancer mortality, and incident type 2 diabetes: a systematic review and dose response meta-analysis. Eur J Epidemiol.

[28] Tremblay MS, Aubert S, Barnes JD (2017). Sedentary Behavior Research Network (SBRN) - terminology consensus project process and outcome. Int J Behav Nutr Phys Act.

[29] Yang L, Cao C, Kantor ED (2019). Trends in sedentary behavior among the US population, 2001-2016. JAMA.

[30] López-Valenciano A, Mayo X, Liguori G, Copeland RJ, Lamb M, Jimenez A (2020). Changes in sedentary behaviour in European Union adults between 2002 and 2017. BMC Public Health.

[31] Allen MS, Walter EE, Swann C (2019). Sedentary behaviour and risk of anxiety: a systematic review and meta-analysis. J Affect Disord.

[32] Stanczykiewicz B, Banik A, Knoll N, Keller J, Hohl DH, Rosińczuk J, Luszczynska A (2019). Sedentary behaviors and anxiety among children, adolescents and adults: a systematic review and meta-analysis. BMC Public Health.

[33] McDowell CP, Dishman RK, Gordon BR, Herring MP (2019). Physical activity and anxiety: a systematic review and meta-analysis of prospective cohort studies. Am J Prev Med.

